# Vanillic Acid Nanocomposite: Synthesis, Characterization Analysis, Antimicrobial, and Anticancer Potentials

**DOI:** 10.3390/molecules29133098

**Published:** 2024-06-28

**Authors:** Baskar Venkidasamy, Umadevi Subramanian, Hesham S. Almoallim, Sulaiman Ali Alharbi, Rahul Raj Chennam Lakshmikumar, Muthu Thiruvengadam

**Affiliations:** 1 Department of Oral and Maxillofacial Surgery, Saveetha Dental College and Hospitals, Saveetha Institute of Medical and Technical Sciences, Saveetha University, Chennai 600077, Tamil Nadu, India; baskarbt07@gmail.com; 2 Department of Periodontics, Saveetha Dental College and Hospitals, Saveetha Institute of Medical and Technical Sciences, Saveetha University, Chennai 600077, Tamil Nadu, India; spumadevi@gmail.com; 3 Department of Oral and Maxillofacial Surgery, College of Dentistry, King Saud University, P.O. Box 60169, Riyadh 11545, Saudi Arabia; hkhalil@ksu.edu.sa; 4 Department of Botany and Microbiology, College of Science, King Saud University, P.O. Box 2455, Riyadh 11451, Saudi Arabia; sharbi@ksu.edu.sa; 5 Department of General Surgery, Saveetha Medical College and Hospitals, Saveetha Institute of Medical and Technical Sciences, Saveetha University, Chennai 602105, Tamil Nadu, India; rahul808@gmail.com; 6 Department of Crop Science, College of Sanghuh Life Science, Konkuk University, Seoul 05029, Republic of Korea

**Keywords:** nanocomposite, vanillic acid, lung cancer, apoptosis, antimicrobes

## Abstract

Recently, nanoparticles have received considerable attention owing to their efficiency in overcoming the limitations of traditional chemotherapeutic drugs. In our study, we synthesized a vanillic acid nanocomposite using both chitosan and silver nanoparticles, tested its efficacy against lung cancer cells, and analyzed its antimicrobial effects. We used several characterization techniques such as ultraviolet–visible spectroscopy (UV-Vis), field emission scanning electron microscopy (FESEM), energy-dispersive X-ray spectroscopy (EDAX), thermogravimetric analysis (TGA), and differential scanning calorimetry (DSC) to determine the stability, morphological characteristics, and properties of the biosynthesized vanillic acid nanocomposites. Furthermore, the vanillic acid nanocomposites were tested for their antimicrobial effects against *Escherichia coli* and *Staphylococcus aureus*, and *Candida albicans*. The data showed that the nanocomposite effectively inhibited microbes, but its efficacy was less than that of the individual silver and chitosan nanoparticles. Moreover, the vanillic acid nanocomposite exhibited anticancer effects by increasing the expression of pro-apoptotic proteins (BAX, Casp3, Casp7, cyt C, and p53) and decreasing the gene expression of Bcl-2. Overall, vanillic acid nanocomposites possess promising potential against microbes, exhibit anticancer effects, and can be effectively used for treating diseases such as cancers and infectious diseases.

## 1. Introduction

According to a GLOBOCAN report, lung cancer is the second most commonly diagnosed type of cancer, followed by breast cancer. Lung cancer is the leading cause of mortality, followed by prostate and colorectal cancers. Moreover, the mortality rate of lung cancer is high in males while lung cancer is the third leading cause of cancer-related deaths in females [1] In the same report, 2,206,771 new lung cancer cases and 1,796,144 lung cancer deaths were reported worldwide in 2020. Approximately 80–90% of lung cancer cases are caused by cigarette smoking; other causes include industrial substances, organic chemicals, air pollution, and radiation exposure. Among the constituents of smoke, benzo(a)pyrene plays a crucial role in lung malignancy by forming DNA adducts [2]. Several diagnostic methods are available for lung cancer including radiography, computed tomography, magnetic resonance imaging, and positron emission tomography [3]. Surgery is the gold-standard treatment for lung cancer. However, surgery is not possible for metastatic and advanced-stage lung cancer. Other treatment methods include radiotherapy (RT), chemotherapy, and immunotherapy [4]. Despite traditional treatment methods, there is increased damage to normal cells, an inability to specifically target tumor cells, and enhanced tumor resistance. Therefore, the search for effective treatment strategies is expanding.

Recently, advances in nanotechnology have received considerable attention owing to their efficiency in overcoming the limitations of traditional chemotherapeutic drugs. Nanoparticles with a diameter of <100 nm are synthesized from polymers, lipids, or metals, including silver and gold, and are used in various medical applications ranging from diagnosis to cancer therapy [5]. Green synthesis is one of the methods used for synthesizing nanoparticles with the help of natural materials that are safe, cost-effective, and eco-friendly [6]. Phytochemical compounds, including polyphenols, sugars, and proteins, play a crucial role as reducing and stabilizing agents during the synthesis of nanoparticles [7]. Vanillic acid is an active dietary phenolic compound found in several edible fruits and plants, including *Amburana cearensis*, *Angelica sinensis*, and green tea, and is an oxidized form of vanillin. It protects biofilms; decreases reactive oxygen species, including hydroxyl radicals and lipid peroxide; and exhibits hepatoprotective, antimicrobial, and anti-inflammatory properties [8]. Vanillic acid suppresses benzo(a) pyrene-induced lung cancer by restoring lysosomal and xenobiotic-metabolizing enzymes, and acts as an anti-proliferative and anti-inflammatory agent [9]. A comparative study was conducted using vanillic acid and vanillic acid silver nanoparticles against CCL_4_-induced hepatotoxicity, and based on biochemical and histological analyses of liver tissue, the authors reported that vanillic acid silver nanoparticles exhibited a more protective effect than vanillic acid alone in a group of CCL_4_-induced rats [10]. In another study, vanillic acid-loaded core–shell gold nanospheres/mesoporous silica nanoparticles exhibited synergistic antibacterial activity against *S. aureus* without cytotoxicity to normal cells [11]. In the current study, we made an attempt to synthesize vanillic acid nanocomposites using chitosan and AgNPs and tested their efficacy against lung cancer cells.

## 2. Results

### 2.1. Characterization of Synthesized Vanillic Acid Nanocomposite

**Figure 1:**
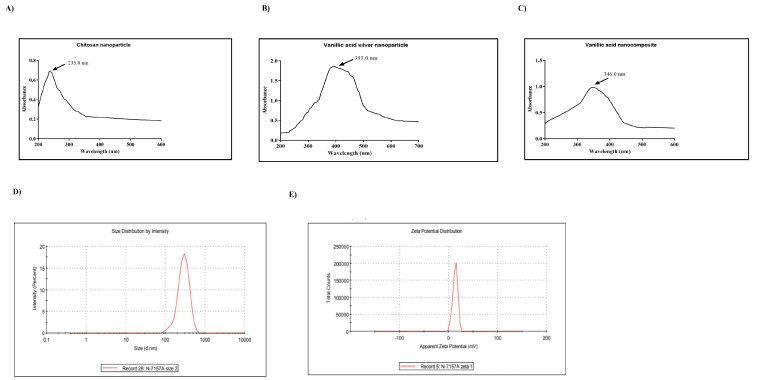
UV-visible spectroscopy and Zetasizer analysis of (**A**) chitosan nanoparticles, (**B**) vanillic acid silver nanoparticle, (**C**) vanillic acid nanocomposite, (**D**) polydispersity index and (**E**) zeta potential.

**Figure 2:**
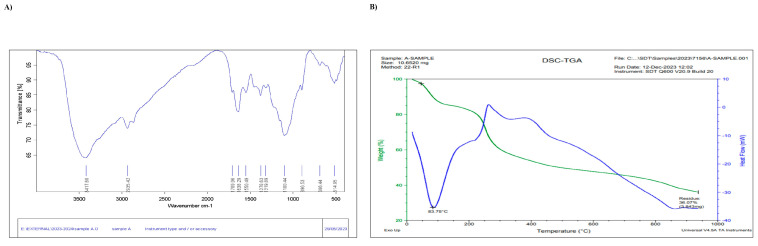
(**A**) Fourier Transform Infrared (FTIR) spectra and (**B**) TGA and DSC thermograms of the vanillic acid nanocomposite.

In our study, the UV-Vis absorbance spectra of the vanillic silver nanoparticles (Figure 1A), chitosan nanoparticles (Figure 1B), and vanillic nanocomposite (Figure 1C) were recorded. The graph shows the peaks of vanillic acid silver nanoparticles at a wavelength of 310 nm, while the vanillic acid nanocomposite showed absorption in the visible range and was shifted to a longer wavelength of 406 nm, which might be due to surface plasmon resonance (SPR). The particle size and size distribution of the nanocomposite were evaluated using a Zetasizer and were found to be 338.4 ± 94.73 nm (Figure 1D), and the particle surface charge (zeta potential) was 13.5 ± 4.96 mV (Figure 1E). FTIR analysis was performed to detect the major functional groups in the vanillin nanocomposite (Figure 2A). The peaks were observed at 3417.68, 2935.42, 1709.96, 1638.29, 1550.49, 1378.63, 1319.89, 1100.44, 896.53, 686.44, 514.95 cm^−1^. The peaks at 514.95, 1100.44, 1638.29, 2935.42, and 3430.76 cm^−1^ indicated the presence of amino, carboxyl, and methyl functional groups, respectively. The intense peak at 1638.29 revealed C=O stretching vibrations (amide I), peaks at 1100.44 and 2935.42 cm^−1^ showed C-H stretching vibrations from the methyl, methylene, and methoxy groups, respectively. The peak at 3417.68 cm^−1^ was attributed to the O-H stretching vibrations in the carboxy moiety.

**Figure 3:**
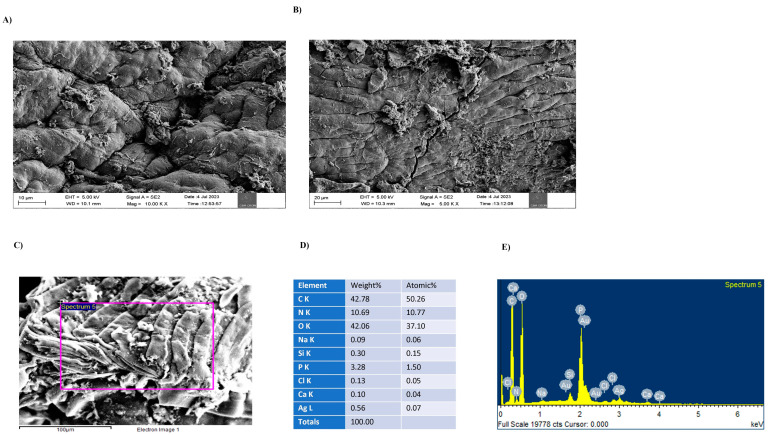
(**A**,**B**). FESEM images of vanillic acid nanocomposites. (**C**–**E**). FESEM images and EDAX analysis of vanillic acid nanocomposite.

Thermal analysis methods measure the properties of a sample and provide information on particle composition, crystallinity, and formation kinetics. Thermogravimetric analysis (TGA) and differential scanning calorimetry (DSC) of the vanillic acid nanocomposites are shown in Figure 2B. The melting point of the vanillic acid nanocomposite was found to be 83.75 °C with a residual loss of 36%. In the TGA analysis, there was an approximately 23% weight loss at 236 °C, which could be due to the dehydration and decomposition of residual chemical compounds, and there was a gradual weight loss up to 1000 °C, after which the weight remained constant. The differential scanning calorimetry results showed a strong endothermic peak at 83.75 °C which indicated that the melting point of the nanocomposite and the exothermic peak at 250 °C and 400 °C could be due to the crystallization of the nanocomposite. Morphological analysis using FESEM showed that the vanillic acid nanocomposite consisted of nanosheets, as shown in Figure 3A,B. FESEM with EDAX analysis of the vanillic acid nanocomposite showed the presence of silver, calcium, carbon, oxygen, nitrogen, and a minor proportion of other elements, such as Si, Cl, and Na, as shown in Figure 3C–E.

### 2.2. Antimicrobial Activities of Vanillic Acid Nanocomposite

**Figure 4:**
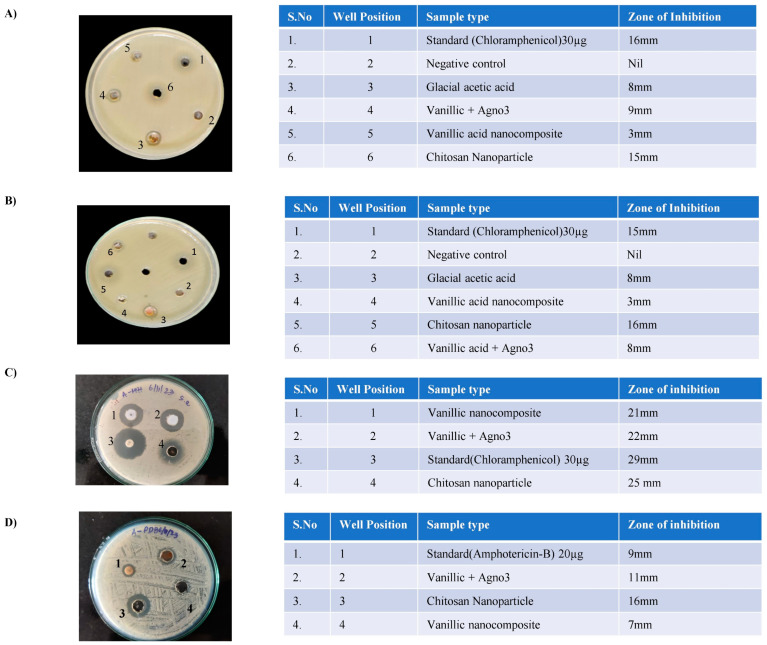
(**A**). Antibacterial activity of vanillic acid nanocomposite against pathogenic *E. coli* (**A**) and its zone of inhibition, and (**B**) non-pathogenic *E. coli* and its zone of inhibition. (**C**). Antibacterial activity of vanillic acid nanocomposite against *S. aureus* and its zone of inhibition and (**D**) fungal species (*C. albicans*) and its zone of inhibition.

The antibacterial effect of vanillic acid nanocomposites was tested against pathogenic *E. coli* (Figure 4A), which revealed that the vanillic acid silver nanoparticles and chitosan nanoparticles exhibited zones of inhibition of 9 mm and 15 mm, respectively, whereas the vanillic acid nanocomposite showed a zone of inhibition of only 3 mm. An examination of non-pathogenic *E. coli* (Figure 4B) revealed that vanillic acid silver nanoparticles and chitosan nanoparticles exhibited zones of inhibition of 8 mm and 16 mm, respectively, whereas the vanillic acid nanocomposite showed a zone of inhibition of only 3 mm. Next, *Staphylococcus aureus* was analyzed (Figure 4C), and it was found that vanillic acid silver nanoparticles and chitosan nanoparticles exhibited zone of inhibition of 22 mm and 25 mm, respectively, while the vanillic acid nanocomposite showed a zone of inhibition of only 21 mm. In *Candida albicans* (Figure 4D), vanillic acid silver nanoparticles and chitosan nanoparticles exhibited zones of inhibition of 11 mm and 16 mm, respectively, whereas the vanillic acid nanocomposite showed a zone of inhibition of only 7 mm. Overall, the vanillic acid nanocomposite exhibited antimicrobial activity; however, the effect was less than that of individual vanillic acid silver nanoparticles and chitosan nanoparticles alone. The effects of individual vanillic acid silver nanoparticles and chitosan nanoparticles were similar to those of the standards (chloramphenicol and amphotericin B).

### 2.3. Effect of Vanillic Acid Nanocomposite on Cell Viability

**Figure 5:**
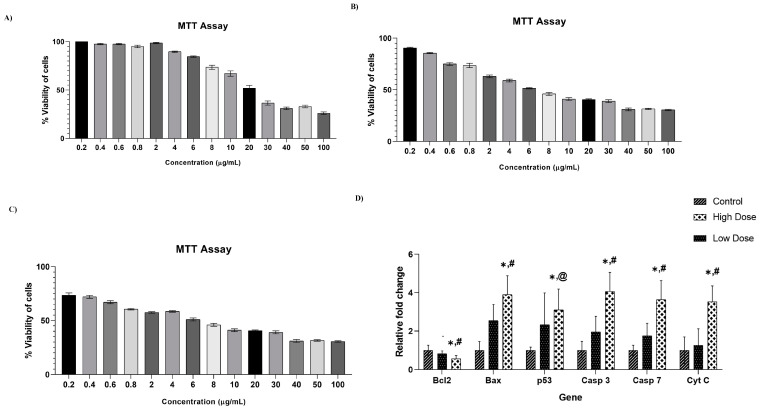
(**A**–**C**) Effect of vanillic acid nanocomposite on cell viability using various tested concentrations on A549 cells after 24 h (**A**), 48 h (**B**), 72 h (**C**) in comparison with control cancer cells (untreated cells). (**D**). Anticancer potential of vanillic acid nanocomposite on apoptotic proteins. Each bar represents the mean ± SD of three independent tests. * Represents that a significant difference occurs between high dose and control; # represents that a significant difference occurs between high dose and low dose; @ represents that no significant difference occurs between high dose and low dose.

The effect of the vanillic acid nanocomposite on cell viability was tested using the MTT assay, and the data are shown in Figure 5A–C. Vanillic acid was treated at different concentrations (0.2, 0.4, 0.6, 0.8, 2, 4, 6, 8, 10.20, 30, 40, 50, 100 μg/mL) and analyzed at three different time intervals (24, 48, and 72 h). The IC50 dose of the vanillic acid nanocomposite at 24 h was 20 μg/mL, while at 48 h and 72 h, it was found to be 6 μg/mL in A549 cells. The results are presented as mean ± SD

### 2.4. Effect of Vanillic Acid Nanocomposite on Apoptosis

Gene expression analysis of apoptotic proteins using RT-PCR was conducted on A549 cells incubated with two doses (6 and 20 μg/mL) of vanillic acid nanocomposites. The levels of pro-apoptotic proteins like caspase 3, 7, Bax, and cytochrome c were significantly increased in vanillic acid nanocomposite-treated lung cancer cells (Figure 5D). On the other hand, the gene expression of Bcl-2 was significantly decreased in vanillic acid nanocomposite-treated A540 cells compared to that in control cancer cells. Interestingly, the expression of the tumor suppressor gene p53 increased in a dose-dependent manner in vanillic acid-treated nanocomposite when compared to control cancer cells, suggesting that the nanocomposite might have stimulated apoptosis via a p53-dependent pathway.

## 3. Discussion

Lung cancer is the leading cause of death worldwide, and chemotherapy is the major treatment strategy. Owing to its limitations, such as side effects in normal cells and the development of cancer resistance, there is a need for effective anticancer agents. In this study, we synthesized vanillic acid nanocomposites using silver and chitosan nanoparticles and characterized them using ultraviolet–visible absorption, dynamic light scattering, FESEM, EDAX, and thermogravimetric analysis. Additionally, our data showed that the vanillic acid nanocomposites exerted antimicrobial and anticancer effects. Our UV-vis data on the nanocomposite showed strong absorption in the visible spectrum, which was higher than that of the silver and chitosan nanoparticles, which ultimately confirmed the surface plasmon resonance phenomenon due to the presence of silver on the nanocomposites [12]. Liu et al. reported that the addition of silver expandsthe absorption wavelength and shifted towards longer wavelengths [13]. Thus, extending the absorption wavelength of the nanocomposite to the visible spectrum showed that the nanocomposite exhibited an increased photocatalytic performance in the visible region. Our Zetasizer data showed that the size was 338.4 ± 94.73 nm. Similar to our study, Keawchaoon and Yoksan reported that an increase in the size of nanoparticles due to the addition of essential oils corresponded to a range of 309–716 nm [14]. Our FTIR analysis showed that there were various peaks at 3417.68, 2935.42, 1709.96, 1638.29, 1550.49, 1378.63, 1319.89, 1100.44, 896.53, 686.44, 514.95 cm^−1^. In a previous study, it was shown that chitosan nanoparticles with an essential oil exhibited a peak at 1644 cm^−1^, which was similar to our results, which exhibited a peak at 1638.29, and suggested that it could be due to electrostatic interactions [15]. Similarly, our spectrum at 3417 cm^−1^ can be attributed to the-OH and -NH groups of chitosan. In a previous study, it was shown that the characteristic band of vanillic acid exhibited at 1530, 1380, 1113, and 1050 cm^−1^ which could be due to the aromatic ring, phenolic hydroxyl group, C–O–C stretching, and (C–C) + (CH) of vanillic acid, respectively, while reports from Stalin and Rajendran are inconsistent with our analysis [16]. Our thermal studies revealed that the nanocomposite exhibited more thermal degradation, and an initial weight loss was observed at a temperature of 46 °C owing to vaporization. In another study, it was found that chitosan and its nanocomposite exhibited weight loss at 75–80 °C [17].

The DSC data analysis revealed the miscibility and compatibility of the components in the nanocomposites. Our data revealed two major peaks and one endothermic peak of the nanocomposite at 83.75 °C which were associated with the evaporation of water, and another major peak around 250 °C which might be due to thermal degradation of the amine units of the nanocomposite. Consistent with our study, the chitosan nanocomposite exhibited a maximum peak at ~88 °C and another peak at ~286 °C, which may be associated with the thermal degradation of the amine units of chitosan [18]. The FESEM analysis of the vanillic acid nanocomposite revealed the morphology of the nanocomposite, which was found to be layered flakes/sheets with a good distribution, similar to those of other studies showing that the graphene nanoparticles were organized into well-distributed sheets [19]. In another study, it was found that a well-layered chitosan matrix exhibited excellent adhesion and intermolecular binding and played a crucial role in improving its physical properties [17]. In our study, vanillic acid nanocomposite preparation was further confirmed by EDX spectroscopy. The EDX spectrum showed the presence of elements in a particular region of the FESEM image. Silver, calcium, O, C, and K were present in the nanocomposite, and there was additional Cl, which may have been due to contamination [20]).

Bacterial cultures of different strains (*S. aureus* and *E. coli*) and fungal strains (*C. albicans*) were inoculated with vanillic nanocomposites, chitosan nanoparticles, and vanillic silver nanoparticles, and the data revealed that all three nanoparticles exhibited antibacterial activity. Similar to our results, several reports have shown that nanocomposites exhibit weak antibacterial activity against Gram-positive and Gram-negative bacteria [21,22]. Studies have revealed that nanomaterials exhibit antibacterial activity owing to their high specific surface area and unique chemical and physical properties [23,24]. The bactericidal effect of nanoparticles could be due to physical damage (destruction of lipid membranes) and chemical damage (oxidative stress). The possible mechanisms for the antibacterial activity of nanocomposites could be (1) direct interaction of the vanillic nanocomposite with the bacterial membrane, (2) the synthesis of reactive oxygen species (superoxide (O_2_·−) and hydroxyl radicals (OH·−), and (3) bacterial membrane damage, leading to the leakage of intracellular materials and electrolytes, and reduced activity of ATPase, ultimately leading to bacterial death [25,26]. The increased generation of free radicals stimulates oxidative damage to DNA and lipids [27]. Another interesting factor that could enhance the antimicrobial potential of nanomaterials is entrapping or enclosing the bacteria, completely separating them from the use of growth medium, and subsequently causing growth inhibition [28].

In our study, we observed that the vanillic acid nanocomposite exhibited anticancer effects by inducing apoptosis in lung cancer cells. The possible mechanisms for the anticancer effect could be (1) diffusion into cancer cells and subsequent pinocytosis, and (2) stimulation of ROS generation, which affects the integrity of DNA and stimulates apoptosis [29,30]. Apoptosis is a mechanism that effectively removes cancer cells by modulating the expression of apoptotic proteins. The BCL2 family of protein is essential for mitochondrial-dependent apoptosis, which involves several proteins, including anti-apoptotic proteins like (Bcl-2 and Bcl-XL) and proapoptotic proteins (BAX, Bim, and Bad) [31]. In our study, we observed that the vanillic acid nanocomposite decreased *Bcl-2* and increased *Bax* expression in lung cancer cells. Alterations in these apoptotic proteins stimulate the release of cytochrome c and induce caspase cascade events that subsequently induce DNA fragmentation and cell death [32]. In our study, we observed that the vanillic acid nanocomposite increased the gene expression of cytochrome c and caspases 3 and 7 in lung cancer cells. Moreover, the tumor suppressor gene, p53, binds to DNA and stimulates p21 expression, which specifically binds to cell cycle complexes to arrest the cell cycle, cell proliferation, and cell division [33]. Our study also enhanced the gene expression of p53 in lung cancer cells treated with vanillic acid nanocomposites.

## 4. Materials and Methods

### 4.1. Chemicals

Chemicals such as chitosan were procured from HI Media Laboratories Pvt. Ltd. (Mumbai, India). Others were procured from Sisco Research Laboratories (SRL) Pvt. Ltd., Mumbai, Maharashtra, India, and Sigma-Aldrich (Pune, India).

### 4.2. Synthesis of Vanillic Acid Nanocomposite

A chitosan (1%) solution was prepared by mixing aqueous acetic acid for 15 min at room temperature, followed by the addition of 5 mL of TPP (0.8%) with constant stirring for 1 h. The obtained chitosan nanoparticle suspension was centrifuged at 12,000 rpm for 10 min and air dried. Separately, vanillic silver nanoparticles were obtained by mixing 10 mL of vanillin with 20 mL of 5 mM silver nitrate solution with constant stirring to obtain a suspension. Chitosan nanoparticles (1 g) and vanillin silver nanoparticle suspension (10 mL) were then used to prepare the nanocomposite and were stirred continuously for 1 h. Following the adding glutaraldehyde, the solution was allowed to settle down overnight and was centrifuged at 12,000 rpm and then the pellet was air dried carefully in a hot air oven, and the resultant vanillic nanocomposite was stored in a refrigerator at 4 °C for further characterization studies.

### 4.3. UV-Visible Spectrophotometry Analysis

The absorbance of the vanillic acid nanocomposite was determined by scanning the wavelength range of 200–800 nm using UV-Vis spectrophotometry (Thermo Fisher Scientific, Waltham, MA, USA), and the highest peak value was obtained.

### 4.4. FTIR Analysis

Fourier transform infrared spectroscopy (FTIR) analysis showed that the vanillic acid nanocomposite was mixed with potassium bromide powder which allowed for the development of a thin disc. The resultant disc sample was then analyzed (400–4000 cm^−1^) using FTIR spectroscopy (Perkin Elmer, Waltham, MA, USA) at a resolution of 1 cm^−1^ to understand the chemical interactions in the nanocomposites.

### 4.5. Zeta Size and Zeta Potential Analyses

The Z-average size and zeta potential were analyzed using a Zetasizer (Malvern Zetasizer, Malvern Worcs, WR14 1XZ, Malvern, UK) to determine the particle size distribution, surface charge, and stability of the vanillic acid nanocomposite.

### 4.6. FESEM

The synthesized vanillin nanocomposites were then subjected to FESEM analysis. The surface morphology, shape, and size distribution of the synthesized vanillic acid nanocomposites were examined using FESEM (SUPRA 55VP instrument, Carl Zeiss, Jena, Germany). It was used to capture FESEM images of the nanocomposite at high resolution under various magnifications. Additionally, FESEM coupled with energy-dispersive X-ray analysis was used to analyze the elemental composition.

### 4.7. TGA-DSC

TGA was used to analyze the mass loss of nanomaterials with respect to different temperature conditions, whereas DSC analysis helped to understand the amount of heat involved in the endothermic/exothermic processes of the nanomaterials. The melting point of the vanillic acid nanocomposites was determined.

### 4.8. Antimicrobial Potential of Vanillic Acid Nanocomposite

The antimicrobial activity of the vanillic acid nanocomposite was determined using the disk diffusion method according to the standard protocol. Nutrient agar medium was prepared by mixing peptone (5.0 g), beef extract (1.5 g), yeast (1.5 g), and sodium chloride (NaCl) 5 g in 1000 mL of distilled water, and the pH was adjusted to 7.4. Finally, the agar (15 g) was mixed and the agar medium was sterilized in a conical flask at a pressure of 150 lbs for 30 min. The medium was then transferred to sterilized Petri dishes in a laminar airflow chamber. Bacterial cultures of different strains (*S. aureus* and *E. coli*) and fungal strains (*C. albicans*) were inoculated with different compositions of 2 mg/mL each well, loaded 40 µL of vanillic nanocomposite, chitosan nanoparticles, vanillic silver nanoparticles (VA + AgNO_3_), and 2% glacial acetic acid and chloramphenicol 30 µg for *Staphylococcus aureus*, nitrofurantoin 300 µg for *E. coli* and amphotericin-B 20 µg for Candida albicans. The plates were placed to observe the growth of the culture, incubated for 24 h at 37 °C, and the zones of inhibition were observed. An overnight culture of each bacterial strain was plated on MHA medium and the fungal strain was plated on potato dextrose agar/broth with 2% glucose.

### 4.9. Cell Lines and Conditions

The lung cancer cell line A549 was purchased from NCCS (Pune, India). The cells were grown in DMEM containing 10 fetal bovine serum (FBS) (10%) (Thermo Fisher Scientific), penicillin (100 units/mL), and streptomycin (100 μg/mL) (Sigma). The cells were then incubated in 5% CO_2_, allowed to attain 80% confluence, and further subcultured according to the manufacturer’s requirements.

### 4.10. MTT Assay

Approximately 6 × 10^3^ cells (A549 cells) were cultured in 96 well plates and treated with vanillic acid nanocomposite (dissolved in 0.1% DMSO) at different doses (0.2, 0.4, 0.6, 0.8, 2, 4, 6, 8, 10, 20, 30, 40, 50, and 100 μg/mL) and analyzed in a dose- and time-dependent manner (24, 48, and 72 h) using MTT (3-[4,5-dimethylthiazol-2-yl]-2,5 diphenyl tetrazolium bromide). The cells were mixed with MTT (20 μL/mL) and incubated for 4 h at 37 °C. Subsequently, 100 μL of 1.0% DMSO was added carefully to each well and allowed to mix for 30 min using a shaker to dissolve the formazan crystals precipitate and analyzed under UV at 595 nm absorbance using a VersaMax ELISA Microplate Reader (Molecular Devices Inc., San Jose, CA, USA).

### 4.11. PCR and Gene Expression

Total RNA was isolated using TRIzol reagent (Takara Bio Inc., Kusatsu, Japan; cat No:9108) according to the standard protocol. For RNA isolation, 1 × 10^7^ of A549 cells were used. A separate cell culture setup was developed, and the cells were incubated with the vanillic acid nanocomposite (2 h incubation for both low and high doses) and RNA isolation was performed. After isolation, the mRNA was quantified using a nanodrop-2000 spectrophotometer (Thermo Fisher Scientific, USA). The reverse transcription procedure (Takara Bio Inc., Japan) was performed using approximately 2 μg of mRNA as a template to produce cDNA, which was then stored at −20 °C. Before being used for RT-PCR, cDNA from each sample was stored at −20 °C. Following the manufacturer’s instructions, a SYBR green kit (Bio-Rad, Hercules, CA, USA #1708882AP) was used to perform quantitative real-time PCR (qRT-PCR) (Bio-Rad). The ΔΔCt method was used to examine the Q-PCR data, and β-actin was used as an internal control to quantify the results in terms of fold change. Primers used in this study are listed in Table 1.

### 4.12. Statistical Analysis

Experiments were performed in triplicate and the results were statistically analyzed using GraphPad Prism version 8 (GraphPad Software, San Diego, CA, USA). One-way analysis of variance (ANOVA) and data are presented as mean ± SD. Differences were considered statistically significant at *p* < 0.05.

## 5. Conclusions

In this study, we synthesized and characterized a vanillic acid nanocomposite and tested its efficacy against microbes and cancer cells. Our data revealed that the vanillic acid nanocomposites exhibited antibacterial and antifungal effects. Vanillic acid nanocomposites stimulate the expression of pro-apoptotic proteins, decrease anti-apoptotic proteins, and ultimately induce apoptosis in lung cancer cells. However, further in vivo anticancer effects of vanillic acid nanocomposites are still lacking, and additional molecular mechanisms that are involved in vanillic acid nanocomposite-treated lung cancer cells are needed.

## Figures and Tables

**Table 1:** List of primers used in this study.

Gene	Forward	Reverse
Caspase-3	5′TTTTTCAGAGGGGATCGTTG3′	5′CGGCCTCCACTGGTATTTTA3′
Caspase-7	5′AGTGACAGGTATGGGCGTTC3′	5′CGGCATTTGTATGGTCCTCT3′
B-Actin	5′CTCTTCCAGCCTTCCTTCCT3′	5′AGCACTGTGTTGGCGTACAG3′
p53	5′TTCCTGAAAACAACGTTCTGTC3′	5′AACCATTGTTCAATATCGTCCG3′
BAX	5′CGAACTGGACAGTAACATGGAG3′	5′CAGTTTGCTGGCAAAGTAGAAA3′
Bcl2	5′GACTTCGCCGAGATGTCCAG3′	5′GAACTCAAAGAAGGCCACAATC3′
Cyt-C	5′CGTTGTGCCAGCGACTAAAAA3′	5′GATTTGGCCCAGTCTTGTGC3′

## Data Availability

Data are contained within the article.
